# Unreliability of the Fixed Bridge

**Published:** 1904-11

**Authors:** 


Unreliability of the Fixed Bridge. —More and more do I wish to emphasize the absolute unreliability of any fixed bridge of more than four teeth, and in some cases even four are too many. We must, therefore, look for some other method of attachment if we do not hark back at once to plate work. Our only hope is in the removable bridge. This is really a reversion to earlier practice, for when the discussion of bridge work pro and con was at the same phase that inlay work now stands, I was among those who doubted its qualifications as a fixture within the oral cavity and made only those that could be removed and cleansed. The first of these (and here I present one of them) was of rubber, with metal ring encircling a gold crown at the back and with clasp metal arms in front. Recently I saw two of these pieces in the same mouth that have been in use fourteen years in excellent condition and doing service. The fact that this method could be used only in the lower jaw somewhat limited its field of service.—*Review.*

